# Depletion‐assisted multiplexed cell‐free RNA sequencing reveals distinct human and microbial signatures in plasma versus extracellular vesicles

**DOI:** 10.1002/ctm2.1760

**Published:** 2024-07-19

**Authors:** Hongke Wang, Qing Zhan, Meng Ning, Hongjie Guo, Qian Wang, Jiuliang Zhao, Pengfei Bao, Shaozhen Xing, Shanwen Chen, Shuai Zuo, Xuefeng Xia, Mengtao Li, Pengyuan Wang, Zhi John Lu

**Affiliations:** ^1^ MOE Key Laboratory of Bioinformatics, Center for Synthetic and Systems Biology, School of Life Sciences Tsinghua University Beijing China; ^2^ Institute for Precision Medicine Tsinghua University Beijing China; ^3^ Geneplus‐Beijing Institute Beijing China; ^4^ Tianjin Third Central Hospital Tianjin China; ^5^ Department of Interventional Radiology and Vascular Surgery Peking University First Hospital Beijing China; ^6^ Department of Rheumatology and Clinical Immunology, Peking Union Medical College, National Clinical Research Center for Dermatologic and Immunologic Diseases (NCRC‐DID), MST State Key Laboratory of Complex Severe and Rare Diseases, MOE Key Laboratory of Rheumatology and Clinical Immunology Peking Union Medical College Hospital, Chinese Academy of Medical Sciences Beijing China; ^7^ School of Life Sciences Peking University–Tsinghua University–National Institute of Biological Sciences Joint Graduate Program, Tsinghua University Beijing China; ^8^ Gastrointestinal Surgery Peking University First Hospital Beijing China

**Keywords:** cell‐free RNA, extracellular vesicles, liquid biopsy, cancer classification

## Abstract

**Background:**

Cell‐free long RNAs in human plasma and extracellular vesicles (EVs) have shown promise as biomarkers in liquid biopsy, despite their fragmented nature.

**Methods:**

To investigate these fragmented cell‐free RNAs (cfRNAs), we developed a cost‐effective cfRNA sequencing method called DETECTOR‐seq (depletion‐assisted multiplexed cell‐free total RNA sequencing). DETECTOR‐seq utilised a meticulously tailored set of customised guide RNAs to remove large amounts of unwanted RNAs (i.e., fragmented ribosomal and mitochondrial RNAs) in human plasma. Early barcoding strategy was implemented to reduce costs and minimise plasma requirements.

**Results:**

Using DETECTOR‐seq, we conducted a comprehensive analysis of cell‐free transcriptomes in both whole human plasma and EVs. Our analysis revealed discernible distributions of RNA types in plasma and EVs. Plasma exhibited pronounced enrichment in structured circular RNAs, tRNAs, Y RNAs and viral RNAs, while EVs showed enrichment in messenger RNAs (mRNAs) and signal recognition particle RNAs (srpRNAs). Functional pathway analysis highlighted RNA splicing‐related ribonucleoproteins (RNPs) and antimicrobial humoral response genes in plasma, while EVs demonstrated enrichment in transcriptional activity, cell migration and antigen receptor‐mediated immune signals. Our study indicates the comparable potential of cfRNAs from whole plasma and EVs in distinguishing cancer patients (i.e., colorectal and lung cancer) from healthy donors. And microbial cfRNAs in plasma showed potential in classifying specific cancer types.

**Conclusions:**

Our comprehensive analysis of total and EV cfRNAs in paired plasma samples provides valuable insights for determining the need for EV purification in cfRNA‐based studies. We envision the cost effectiveness and efficiency of DETECTOR‐seq will empower transcriptome‐wide investigations in the fields of cfRNAs and liquid biopsy.

**Keypoints:**

- DETECTOR‐seq (depletion‐assisted multiplexed cell‐free total RNA sequencing) enabled efficient and specific depletion of sequences derived from fragmented ribosomal and mitochondrial RNAs in plasma.
- Distinct human and microbial cell‐free RNA (cfRNA) signatures in whole Plasma versus extracellular vesicles (EVs) were revealed.
- Both Plasma and EV cfRNAs were capable of distinguishing cancer patients from normal individuals, while microbial RNAs in Plasma cfRNAs enabled better classification of cancer types than EV cfRNAs.

## Graphical abstract

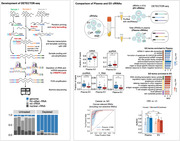

## 1 INTRODUCTION

In recent years, liquid biopsy has emerged as a non‐invasive approach for assessing circulating biomarkers in various body fluids, enabling the monitoring of physiologic and disease states.^1^ Cell‐free RNAs (cfRNAs), given their virtue of being highly dynamic, hold great potential to reflect the pathophysiological processes, thus offering unique opportunities for disease monitoring. Previous reports have suggested that cfRNAs are packaged into various extracellular complexes, such as extracellular vesicles (EVs, including microvesicles and exosomes) and non‐vesicular ribonucleoproteins (RNPs).^2^ Due to the protection of EV, RNA binding proteins (RBPs) and/or their self‐structures, cfRNAs are capable of being stably present in human bloodstream.^3^ While previous studies have predominantly focused on total cfRNAs^4^, ^5^, ^6^ or EV^7^, ^8^, ^9^ cfRNAs in plasma, the transcriptional differences between these two entities remain poorly understood.

Efforts to characterise cfRNAs initially centred around small RNAs like microRNAs (miRNAs) because of the nature of RNA degradation and fragmentation in biofluids. However, miRNAs represent only a small proportion of the human transcriptome.^10^ Consequently, investigations have expanded to encompass a broader range of cfRNA species, including messenger RNAs (mRNAs), long non‐coding RNAs (lncRNAs) and circular RNAs (circRNAs).^4^, ^5^, ^6^, ^7^, ^11^ These cell‐free long RNA species (>50 nt) have relatively low concentrations in human blood due to the presence of RNases, and they are typically fragmented (∼50–200 nucleotides) with incomplete RNA ends.^12^ Conventional small RNA‐seq approaches, which rely on ligating sequencing adapters based on the 5′ phosphate (5′ P) and 3′ hydroxyl (3′ OH) ends of RNA, are inadequate for analysing these fragmented cfRNAs.^13^

Recently, several sequencing approaches have been developed to profile cell‐free long RNA fragments in plasma or EVs. Phospho‐RNA‐seq integrates T4 polynucleotide kinase into ligation‐based TruSeq small RNA‐seq, enabling the recovery of mRNA and lncRNA fragments lacking 5′ P and/or 3′ OH ends. However, the libraries generated by phospho‐RNA‐seq contain high proportions of ribosomal RNAs (rRNAs) and Y RNAs, limiting the capacity to detect other informative RNA species.^12^ Another method, SILVER‐seq, captures both small and long cfRNAs from extremely low‐input serum samples.^14^ However, substantial DNA contamination seemed to be an issue of SILVER‐seq.^15^ Recently, SMARTer stranded total RNA‐seq (hereafter called SMARTer‐seq) has been employed in several cfRNA studies,^4^, ^5^, ^6^, ^7^ utilising a proprietary ZapR and R‐probes to deplete unwanted ribosomal sequences.^16^, ^17^ However, as a commercial kit, SMARTer‐seq is not specifically optimised for cfRNA library preparation from plasma and is cost‐inefficient. Overall, the current cfRNA sequencing approaches were hindered by unwanted RNAs, DNA contamination and high cost.

Current targeted depletion strategies for unwanted RNAs, such as RiboMinus kits (Thermo Fisher Scientific), Ribo‐Zero technology (Illumina) and RNase H‐mediated digestion of RNA:DNA hybrids,^16^ primarily operate at the RNA level and require relatively intact RNA molecules. Consequently, these methods are unsuitable for low‐input and fragmented cfRNA samples. In contrast, the Cas9‐mediated targeted DNA cleavage technique, also known as DASH (depletion of abundant sequences by hybridisation),^18^ provides the capability to selectively cleave complementary DNA (cDNA) molecules derived from rRNAs during the double‐stranded DNA stage after library amplification. Notably, this method only requires the design of a set of specific single‐stranded guide RNAs (sgRNAs) to direct Cas9 cleavage of undesirable sequences. Therefore, CRISPR‐Cas9 presents a highly advantageous approach for the targeted removal of over‐represented sequences in the libraries of low‐input and fragmented cfRNA samples derived from plasma and EVs.

In this study, we present an optimised cfRNA sequencing method, DETECTOR‐seq (depletion‐assisted multiplexed cell‐free total RNA sequencing), which utilises early barcoding and CRISPR‐Cas9 to reduce costs and deplete highly abundant, fragmented rRNAs and mitochondrial RNAs (mtRNAs) in human plasma. Subsequently, we used DETECTOR‐seq to investigate 113 plasma cfRNA samples (including 61 plasma total RNA and 52 EV RNA libraries), derived from healthy donors, lung cancer (LC) and colorectal cancer (CRC) patients. To the best of our knowledge, this study is the first to compare paired total and EV‐selected transcriptomes in the same plasma samples, suggesting their distinct signatures and different utilities in cancer liquid biopsy.

## 2 RESULTS

### 2.1 Development of DETECTOR‐seq to profile cell‐free transcriptome

**FIGURE 1:**
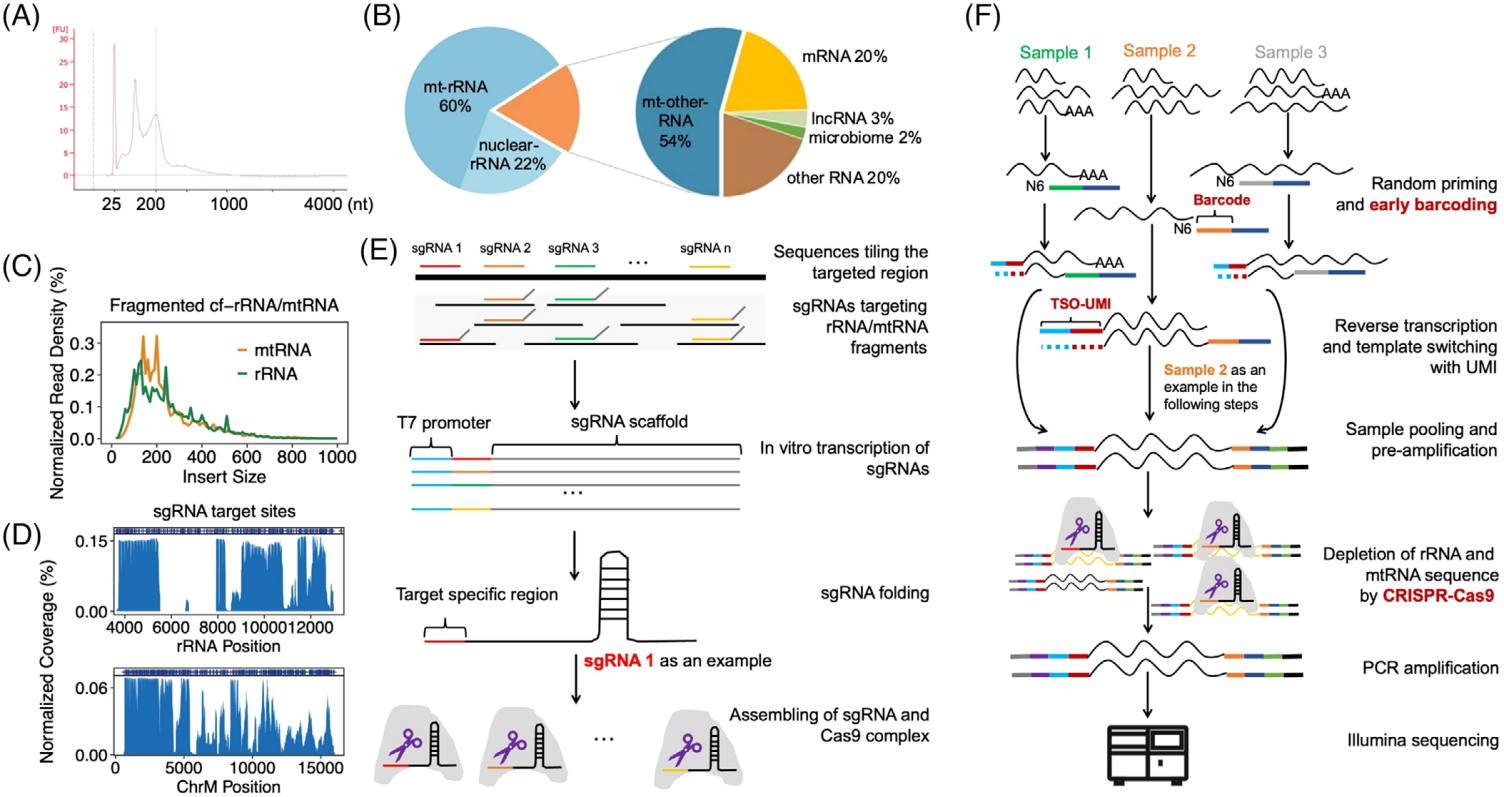
Depletion‐assisted multiplexed cell‐free total RNA sequencing (DETECTOR‐seq). (A) Bioanalyzer trace of cell‐free RNA (cfRNA) fragment lengths in a human plasma sample. (B) The relative proportion of reads for various RNA biotypes detected by total RNA sequencing averaged by three human plasma samples. (C) Distribution of reads’ insert size for the fragmented rRNAs and mtRNAs, derived from the above sequencing data. (D) Distribution of reads’ coverage. Blue bars on top represent single‐stranded guide RNA (sgRNA) target sites. (E) The designed sgRNAs tiling the fragmented rRNA and mtRNA sequences. (F) Schematic overview of DETECTOR‐seq workflow. First, cfRNAs are reverse transcribed with random primers and TSO. Sample barcodes and UMIs are introduced during this step. Second, after calibrating input amounts, samples are pooled and pre‐amplified. Third, complementary DNAs (cDNAs) of rRNAs and mtRNAs are depleted by CRISPR‐Cas9. Subsequently, DETECTOR‐seq library is further amplified, then sequenced on an Illumina platform. mtRNA, mitochondrial RNA; rRNA, ribosomal RNA; TSO, template‐switching oligo; UMI, unique molecular identifier.

The sequencing of cfRNAs in plasma and EVs usually meets the following obstacles. First, consistent with previous reports,^10^ we observed that plasma cfRNAs were degraded with a fragment length of <200 nucleotides (Figure 1A). These fragmented cfRNAs are hard to be detected by RNA‐seq protocols based on ligation techniques requiring intact RNA ends. Second, rRNAs and mtRNAs accounted for ∼92% of all clean reads (reads after removing adapters and filtering low‐quality reads), while mRNAs and lncRNAs collectively made up only a small fraction (∼4%) of cell‐free transcriptome (Figure 1B). It is worth noting that microbe‐derived RNAs can also be detected in human plasma with a relatively small fraction (∼.4%; Figure 1B). The high fractions of rRNAs and mtRNAs hamper the detection of other informative RNA species. And they are fragmented into pieces in plasma, making them hard to be removed (Figure 1C,D). Third, cfRNAs are usually in the range of hundred picograms to several nanograms per mL of human plasma,^14^ which can be easily lost and contaminated during purification and amplification. Furthermore, low cfRNA input usually requires 20–24 polymerase chain reaction (PCR) amplification cycles for library preparation, which produces a high duplication ratio of raw reads. Meanwhile, DNA contamination ignorable in conventional RNA‐seq is often over‐amplified, causing a big issue in cfRNA‐seq.^15^

To improve the efficiency and reliability of cfRNA detection, we developed DETECTOR‐seq to profile cell‐free transcriptome in human plasma (Figure 1E,F). DETECTOR‐seq captures fragmented cfRNAs with unbiased random priming and template switching. Then, we adapted and modified a previously described method termed CRISPR/Cas9–based DASH^18^ to remove the abundant sequences derived from ribosomal and mitochondrial RNAs in the cDNA library. In this step, guide RNAs (sgRNAs) in the CRISPR‐Cas9 are specifically optimised for human plasma cfRNAs (Supporting Information Figures 1 and 2), covering the fragmented rRNA and mtRNA sequences (Figure 1D,E). The sgRNAs are in vitro transcribed using T7 RNA polymerase, then bind with Cas9 nuclease to form RNP complex and induce site‐specific cleavage with the endonuclease activity of Cas9 (Figure 1E), thus preventing further amplification of cDNAs derived from rRNAs and mtRNAs in the final sequencing library. Meanwhile, DETECTOR‐seq utilises early barcoding during reverse transcription. The multiplexed library will cope with low content of plasma cfRNAs and reduce experimental time and cost as well. It is also worth mentioning that unique molecular identifiers (UMIs) are added to every sequence in the reverse transcription step, hence DETECTOR‐seq is capable of removing PCR duplicates to avoid RNA quantification bias. In addition, we also optimised cfRNA extraction (Supporting Information Figure 3) and residual DNA digestion (Supporting Information Figure 4) protocols.

### 2.2 Analytical validation demonstrating superior performance of DETECTOR‐seq

**FIGURE 2:**
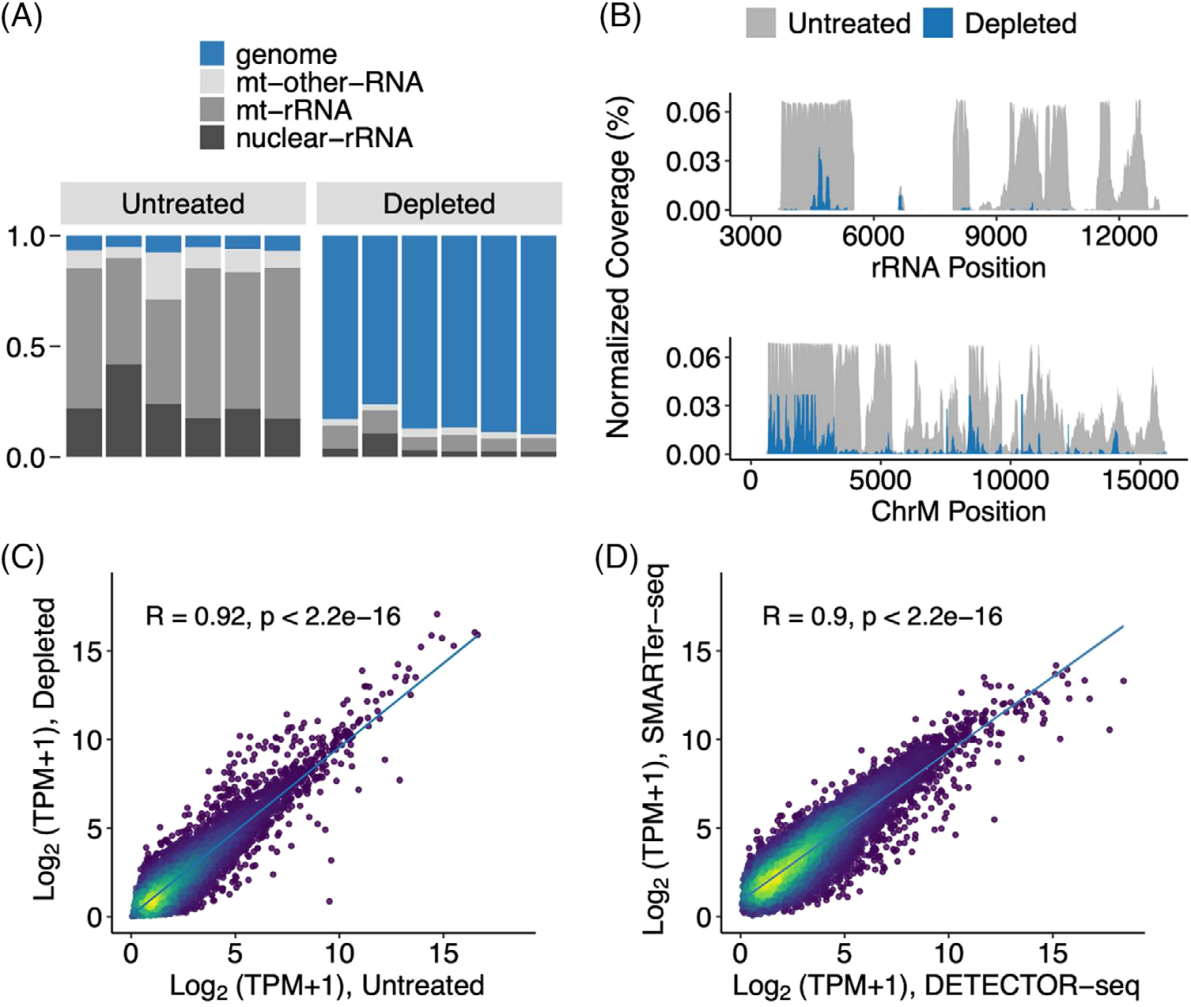
Efficient and specific depletion of ribosomal RNA (rRNA) and mitochondrial RNA (mtRNA) sequences. (A) The read distributions and (B) coverages of untreated and rRNA/mtRNA‐depleted depletion‐assisted multiplexed cell‐free total RNA sequencing (DETECTOR‐seq) libraries. The ‘genome’ category in A represented that reads did not align to ribosomal and mitochondrial RNAs but were successfully aligned to the human genome. Read coverage was normalised to total mapped reads. Pearson correlation of cell‐free RNA (cfRNA) expression levels between (C) untreated and rRNA/mtRNA‐depleted DETECTOR‐seq libraries, and (D) DETECTOR‐seq versus SMARTer‐seq. TPM, transcripts per million mapped reads (rRNA/mtRNA reads were removed).

To examine whether DETECTOR‐seq can deplete the unwanted rRNA and mtRNA sequences effectively and specifically, we split a single plasma sample into two equal aliquots for experimental conditions of untreated versus depleted, with six biological replicates. In the untreated samples, reads mapped to rRNAs and mtRNAs collectively represented ∼94% of all mapped reads. After CRISPR‐Cas9 treatment, these unwanted sequences were decreased to only ∼15% of mapped reads, only about one‐sixth of the untreated ones (Figure 2A). By comparing untreated and depleted aliquots, we observed evident decreases in the normalised coverage of rRNAs and mtRNAs (Figure 2B). Meanwhile, the expression levels of detected genes other than rRNAs and mtRNAs between the untreated and depleted aliquots were well correlated, indicating minimal off‐target effect (Pearson correlation, *R* = .92, *p* value <2.2×^−16^; Figure 2C). It is worth noting that although off‐target effects are minimal, the abundance levels of certain pseudogenes, Y RNAs and lncRNAs may still be influenced by the depletion treatment. We have provided detailed annotations in Supporting Information Figure 2 regarding the RNAs that could potentially be affected by off‐target effects. By comparing the cfRNA expression profiles obtained from DETECTOR‐seq and SMARTer‐seq, we found that the expression levels of detected genes using these two methods were also well correlated (Pearson correlation, *R* = .90, *p* value <2.2×^−16^; Figure 2D). In summary, the above results demonstrate the efficient and specific depletion of unwanted sequences in DETECTOR‐seq.

To further evaluate the performance of DETECTOR‐seq, we prepared cfRNA libraries in a 3‐plex, 4‐plex or 5‐plex manner determined by RNA concentrations. The total read numbers of different barcoded samples in a single multiplexed pool were relatively uniform, varying less than 1.5‐fold in the 3‐ and 4‐plex samples and less than threefold in the 5‐plex samples (Supporting Information Figure 5A). In addition, the UMI strategy in DETECTOR‐seq retained significantly more reads than the non‐UMI approach after duplicated reads were removed (Supporting Information Figure 5B). And a sharp edge of reads’ distribution across exon–intron splice junctions suggested that the majority of DNA contamination was effectively removed (Supporting Information Figure 5C). To evaluate the impact of plasma input volume on the number of detected genes, we utilised five plasma samples from different individuals, with each plasma sample divided into aliquots of 200, 400, 600, 800 and 1000 µL, and subjected to DETECOR‐seq, respectively. Around 4000 genes were detected with the minimum (i.e., 200 µL) volume. The detected gene number linearly increased until a plateau between 800 and 1000 µL, suggesting the detected genes would be saturated after 1 mL of plasma (Supporting Information Figure 5D). While highly correlated cfRNA expression levels were observed within technical triplicates (R1–R3), the correlations were slightly decreased between biological triplicates (N1–N3; Supporting Information Figure 5E). Furthermore, based on External RNA Controls Consortium (ERCC) RNA Spike‐In Mix, we found a high correlation between expected and observed levels of transcript abundance (Pearson correlation, *R* = .91, *p* value <2.2×^−16^; Supporting Information Figure 5F). These results not only demonstrate DETECTOR‐seq's high accuracy and reproducibility but also suggest its capability of capturing subtle differences in cfRNA profiles between different individuals.

Then, we randomly subsampled a dataset (*n* = 24) of DETECTOR‐seq for saturation analyses of detected UMIs (transcripts) and genes. Although the detected UMIs kept increasing when more reads in 1 mL plasma were sequenced (Supporting Information Figure 5G), the detected gene numbers were quickly saturated at approximately 5 million genome‐aligned reads (Supporting Information Figure 5H). These results indicate that DETECTOR‐seq achieves saturation of cfRNA detection at a low sequencing depth.

### 2.3 Better contamination control and cost effectiveness of DETECTOR‐seq than other cfRNA‐seq methods

**FIGURE 3:**
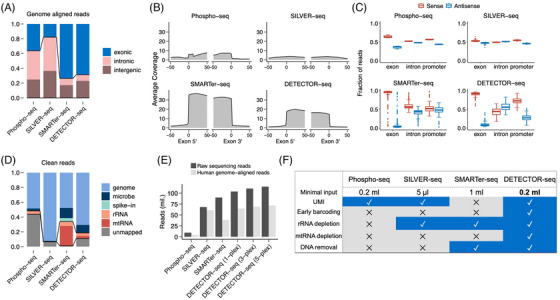
Comparing depletion‐assisted multiplexed cell‐free total RNA sequencing (DETECTOR‐seq) with other cell‐free RNA (cfRNA)‐seq methods. (A) Average percentages of genome‐aligned reads mapping to exonic, intronic and intergenic regions for four different cfRNA‐seq methods. (B) Average coverage across all mRNAs’ 5′ and 3′ exon boundary sites flanking upstream and downstream by 50 bp. (C) Average percentages of reads located in the sense and antisense strands of mRNAs’ exons, introns and promoters. (D) Average percentages of clean reads (after trimming low‐quality and adapter sequences) assigned to different sources. (E) Numbers of raw sequencing reads and human genome‐aligned reads with a fixed budget of $300 for each method. (F) Summary of key techniques used in the four cfRNA‐seq approaches. Numbers of used samples: phospho‐seq: 15; SILVER‐seq: 128; SMARTer‐seq: 373; DETECTOR‐seq: 113.

We benchmarked the performance of DETECTOR‐seq compared to three other cfRNA‐seq methods, including phospho‐RNA‐seq,^12^ SILVER‐seq^14^ and SMARTer‐seq.^19^ The number of samples used in the comparison was listed in Supporting Information Table 5. Within the total genome‐aligned reads, DETECTOR‐seq and SMARTer‐seq had comparable ratios of exonic reads (∼70%), while those of SILVER‐seq and phospho‐RNA‐seq were under 40% (Figure 3A). The lower ratio of exonic reads for SILVER‐seq was presumably due to severe DNA contamination according to a previous report.^15^ We also visualised the read coverage across exon boundary sites flanked upstream and downstream by 50 bp, where DETECTOR‐seq and SMARTer‐seq showed more evident decreases of read coverage from exon to intron/intergenic region than SILVER‐seq and phospho‐RNA‐seq (Figure 3B). As far as we know, all of the four cfRNA‐seq methods should preserve the strand specificity of RNAs. Thus, the enrichment of exons’ sense over antisense reads of DETECTOR‐seq and SMARTer‐seq further confirmed their reads’ quality (Figure 3C). The above results demonstrate that DETECTOR‐seq and SMARTer‐seq have better DNA contamination control than SILVER‐seq. It was worth noting that phospho‐RNA‐seq was developed from a small RNA‐seq method, and the read coverage across exon boundary sites and the enrichment of exons’ sense over antisense reads may be affected by the read distribution of small RNAs.

In addition, we showed that DETECTOR‐seq displayed a higher ratio of reads mapped to human genome (∼71%) than those of SMARTer‐seq (∼48%) because DETECTOR‐seq removed mitochondrial RNAs more efficiently than SMARTer‐seq (Figure 3D). Furthermore, because of its early barcoding and multiplexing strategy, DETECTOR‐seq can produce more raw reads and genome‐aligned reads than the other cfRNA‐seq approaches (Figure 3E, Supporting Information Figure 6). Cost details were explained in Supporting Information Tables 6 and 7. Overall, by summarising and comparing key characteristics of these approaches (Figure 3F), we collectively demonstrate that DETECTOR‐seq has better contamination control and more efficient cost than the other cfRNA‐seq methods.

### 2.4 Distinct human and microbial RNA signatures in plasma versus extracellular vesicles

**FIGURE 4:**
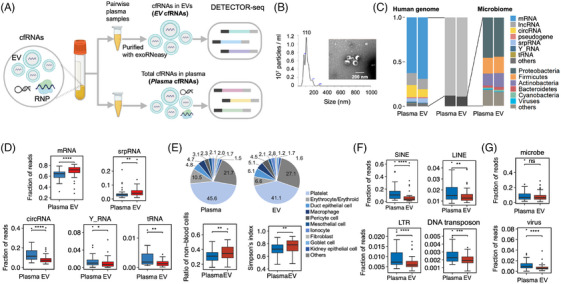
Distinct human and microbial RNA signatures in *Plasma* versus extracellular vesicle (*EV*). (A) Illustration of sequencing *Plasma* cell‐free RNAs (cfRNAs) and *EV* cfRNAs in paired plasma samples. (B) Plasma EVs were characterised by nanoparticle tracking analysis and transmission electron microscopy (scale bar represents 200 nm). (C) Distribution of reads mapped to human genome and microbiome in *Plasma* and *EV* cfRNA datasets. Left: RNA spectrum mapping to human genome. The genome mapped reads were sequentially assigned to mRNA, lncRNA, pseudogene, tRNA, srpRNA, snoRNA, snRNA, Y_RNA, misc_RNA and other exon regions with HTSeq according to GENCODEv38 annotation. Others include snoRNA, snRNA, misc_RNA and other exon regions. Right: relative abundance of reads aligned to different phyla. (D) Differential human RNA species between *Plasma* and *EV* cfRNAs. (E) Pie charts show the average fractional contributions of various cell types to the *Plasma* and *EV* transcriptomes. Box plots show the diversity of cell type contributions to the *Plasma* and *EV* transcriptomes measured by the ratio of non‐blood cells and Simpson's index. (F) Boxplots represent the enrichment of cfRNAs derived from transposable elements in *Plasma* cfRNAs compared to *EV* cfRNAs. The different transposable element categories, including short interspersed elements (SINEs), long interspersed elements (LINEs), LINEs with long terminal repeats (LTRs) and DNA transposons, are represented. (G) The fractions of reads aligned to microbe and virus. *Plasma*: 44 samples; *EV*: 44 samples (all samples paired). *p value <.05, **p value <.01, ****p value <.0001, Wilcoxon rank sum test, two‐tailed.

Subsequently, we employed DETECTOR‐seq to conduct pairwise investigations of total cfRNAs and EV cfRNAs in human plasma (Figure 4A). A proportion of cfRNAs are enclosed inside EVs such as MVs and exosomes.^20^ Meanwhile, it is also reported that a significant proportion of cfRNAs are not within EVs but associated with proteins to form non‐vesicular RNPs.^21^ Although both plasma total cfRNAs^4^, ^5^, ^6^ and EV cfRNAs^7^, ^9^ have been used in liquid biopsy studies, a pairwise comparison of their distinct signatures and utilities has not been conducted yet.

In this study, a total of 139 plasma cfRNA samples were sequenced, which included samples obtained from healthy donors as well as patients with LC and CRC (Supporting Information Figure 7 and Supporting Information Table 10). EVs were purified using a membrane‐affinity column, concentrating particles predominantly within the size range of 50–200 nm, with a peak around 110 nm. Morphological examination using transmission electron microscopy (TEM) confirmed the presence of the characteristic cup‐shaped structure commonly associated with EVs (Figure 4B). After conducting quality control (QC) procedures on the RNA samples and sequencing data, a total of 113 datasets passed the QC criteria (Supporting Information Figures 7–9). Out of these 113 datasets, 61 were derived from total cfRNA‐seq of plasma, while 52 were obtained from EV cfRNA‐seq of plasma. Among them, 44 datasets were paired, meaning they originated from the same plasma samples. In the following description, total cfRNA‐seq of plasma and EV cfRNA‐seq of plasma will be abbreviated to *Plasma* cfRNA and *EV* cfRNA, respectively.

From a general view, there was a high degree of similarity between *Plasma* and *EV* cfRNAs, with ∼90% of aligned reads mapping to human genome and ∼10% mapping to microbe genomes (Figure 4C). For human cfRNAs, mRNA, lncRNA and circRNA were the major RNA types. For microbial cfRNAs, the most abundant phylum was *Proteobacteria*, followed by *Firmicutes* and *Actinobacteria*. The human and microbial RNA compositions resembled previous reports.^19^, ^22^

In addition, distinctive signatures were revealed for the first time by our pairwise comparison between *Plasma* (*n* = 44) and *EV* (*n* = 44) cfRNAs (all samples were paired). We first observed that *Plasma* cfRNAs had more short fragments (20–100 nt), while *EV* cfRNAs had more long fragments (>100 nt; Supporting Information Figure 10). We also observed that structured tRNAs, Y RNAs and circRNAs were enriched in *Plasma* cfRNAs, while mRNAs and signal recognition particle RNAs (srpRNAs) were enriched in *EV* cfRNAs (Figure 4D). These findings align with a previous study that reported a significant enrichment of tRNA and Y RNA fragments in extracellular RNPs.^2^ Moreover, we also found that the relative abundance of circRNAs was slightly higher in *Plasma* cfRNAs than *EV* cfRNAs (median 13.6% vs. 8.8%, *p* value <.0001, Wilcoxon rank sum test; Figure 4D and Supporting Information Figure 11), perhaps due to its circle‐like structure resisting degradation outside EVs. We totally identified 13 circRNAs differentially enriched in *Plasma* versus *EV* cfRNAs. Only one of them, hsa_circ_0048555, was enriched in EVs (Supporting Information Figure 12). Reads mapped to the back‐spliced junction were used to calculate the enrichment.

A recent study provided a framework to infer cell types of origin of the cell‐free transcriptome.^23^ We utilised this method and found a high similarity of the cell types of origin between *Plasma* and *EV* transcriptomes (Figure 4E). Platelets and erythrocytes were inferred as the major origins for both *Plasma* and *EV* cfRNAs, which was in agreement with the previous study.^23^ Intriguingly, we found non‐blood cells contributed more to *EV* cfRNAs than to *Plasma* cfRNAs (*p* value <.01, Wilcoxon rank sum test; Figure 4E). Therefore, the diversities of cell types of origin (measured by Simpson's index) of *EV* cfRNAs were slightly higher than those of *Plasma* cfRNAs (.75 vs. .70, *p* value <.01, Wilcoxon rank sum test; Figure 4E).

A noteworthy discovery has been made regarding the presence of RNAs originating from transposable elements (TEs) and other repetitive elements in the cell‐free transcriptome.^24^ In our current investigation, we provide evidence demonstrating a significant enrichment of cfRNAs derived from TEs in *Plasma* cfRNAs compared to *EV* cfRNAs. These TEs include short interspersed elements (SINEs), long interspersed elements (LINEs), LINEs with long terminal repeats (LTRs) and DNA transposons (Figure 4F).

We also identified distinct microbe genera in *Plasma* and *EV* cfRNAs (Supporting Information Figure 13). While there was no significant difference in the ratio of microbial reads between *Plasma* and *EV* cfRNAs, we did observe a significant increase in cfRNAs mapped to viral genomes in *Plasma* cfRNAs (Figure 4G). Meanwhile, viruses such as *Senecavirus*, *Cheravirus*, *Orthopoxvirus*, *Tenuivirus* and *Rhadinovirus* were enriched in *Plasma* cfRNAs, while *Intestinimonas*, *Mordavella* and *Jonquetella* were enriched in *EV* cfRNAs (Supporting Information Figure 13). In summary, the above comparison results have revealed distinct molecular characteristics between *Plasma* and *EV* cfRNAs in terms of fragment size, RNA species, cell types of origin, TE RNAs and microbe genera.

### 2.5 Functional roles and sequence motifs of selective *Plasma* and *EV* cfRNAs

**FIGURE 5:**
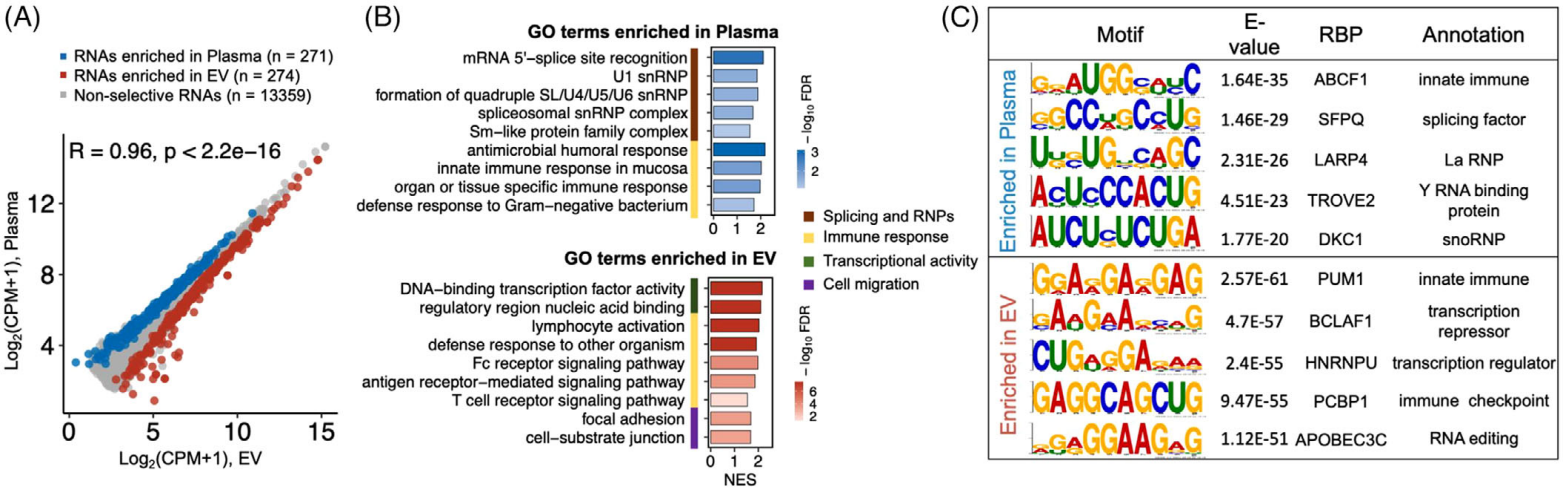
Distinct Gene Ontology (GO) terms, motifs and binding proteins of the selective *Plasma* and extracellular vesicle (*EV*) cell‐free RNAs (cfRNAs). (A) Definition of the selective cfRNAs enriched in *Plasma* or *EV*. Cut‐off: |fold‐change| >1 and false discovery rate (FDR) <.1. (B) Top enriched GO terms of the selective cfRNAs. The *x*‐axis represents the normalised enrichment score (NES), which indicates the relative enrichment of the GO terms, and the colours filled in the bars represent the *p* values adjusted by Benjamini–Hochberg method. (C) Top enriched motifs and their corresponding RNA binding proteins (RBPs) of the selective cfRNAs. *Plasma*: 44 samples; *EV*: 44 samples (all samples paired).

To find selective functions and motifs of cfRNAs in EVs, we identified 545 selectively distributed RNAs showing significantly differential abundance between *Plasma* and *EV* transcriptomes (|fold‐change| >1 and false discovery rate [FDR] <.1; Figure 5A and Supporting Information Figure 14). Among them, 271 cfRNAs were enriched in *Plasma*, while 274 cfRNAs were enriched in *EVs*. We investigated the functional roles and biological pathways of these selective cfRNAs (Figure 5B and Supporting Information Figure 14). Based on functional enrichment analysis, we found that the selective RNAs elevated in *Plasma* were significantly enriched in terms associated with RNA splicing, RNP (e.g., mRNA 5′ splice site recognition, U1 snRNP, spliceosomal snRNP complex and Sm‐like protein family complex), antimicrobial and innate immune responses. Meanwhile, the selective RNAs that were enriched in *EVs* were primarily associated with DNA binding transcription factor activity, focal adhesion, cell–substrate junction and T cell receptor signalling immune pathway. Notably, we also found different immune pathways enriched in the selective cfRNAs of *Plasma* versus *EVs* (Figure 5B and Supporting Information Figure 15). The organ or tissue‐specific immune response and antimicrobial humoral response immune signalling pathways are enriched in *Plasma* cfRNAs, while defence response to other organisms, Fc receptor signalling pathway, T cell receptor signalling pathway are enriched in *EV* cfRNAs (Supporting Information Figure 15).

We further investigated sequence motifs and their associated RBPs for the selective cfRNAs (Figure 5C and Supporting Information Figure 16). And we found that the selective cfRNAs enriched in *Plasma* contained binding motifs/sites for ABCF1, a protein that plays a role in innate immune response^25^; SFPQ, a splicing factor; LARP4, a La RNP; TROVE2, a Y RBP; and DKC1, a snoRNP. Meanwhile, the selective cfRNAs enriched in *EVs* contained binding motifs/sites for PUM1, a protein that participates in human innate immune response^26^; BCLAF1, a transcription factor; HNRNPU, a transcription suppressor; PCBP1, a previously reported immune checkpoint^27^; APOBEC3C, an RNA editing enzyme. These enriched motifs and their associated RBPs were consistent with the biological functions of the selective cfRNAs revealed above.

### 2.6 Specific cancer‐related signals in *Plasma* and *EV* cfRNAs

**FIGURE 6:**
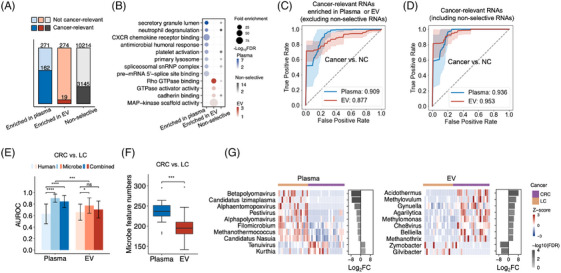
Cancer‐relevant cell‐free RNA (cfRNA) signatures in *Plasma* and extracellular vesicle (*EV*). (A) Cancer‐relevant ones (differentially expressed between cancer patients and normal controls, |log_2_fold‐change| >1 and FDR <.05) in the selective and non‐selective human cfRNAs. Cancer: colorectal cancer (CRC) and lung cancer (LC); NC: normal control. (B) Enriched Gene Ontology (GO) terms related to cancer‐relevant human cfRNAs. The colours filled in the points represent the *p* values adjusted by the Benjamini–Hochberg method. The point size represents the fold enrichment of gene set with geneRatio/bgRatio. Performances (average of 20 bootstrap procedures) of cancer‐relevant human cfRNAs distinguishing cancer patients from normal controls when excluding (C) and including (D) non‐selective cfRNAs. (E) The area under the receiver operating characteristic curves (AUROCs) of cancer type classification (CRC vs. LC) using human‐ or microbe‐derived reads in *Plasma* and *EV* cfRNAs. (F) Numbers of microbial features (genus) with significantly differential abundance (|log_2_fold‐change| >1 and FDR <.1) between CRC and LC in 20 bootstrap procedures. (G) Distinct cancer‐type‐specific microbial features (genus) identified in *Plasma* and *EV* cfRNAs. Heatmaps show *z*‐scores of the abundance levels of these microbial RNA features; bar plots illustrate their average log_2_FCs and FDRs between CRC and LC. FC, fold‐change; FDR, false discovery rate. *p value <.05, ***p value <.001, ****p value <.0001, Wilcoxon rank sum test, two‐tailed. CRC samples: *Plasma* (*n* = 23), *EV* (*n* = 19), 19 of them paired; LC samples: *Plasma* (*n* = 19), *EV* (*n* = 19), 18 of them paired; NC samples: *Plasma* (*n* = 19), *EV* (*n* = 14), seven of them paired.

Next, we compared the potential of *Plasma* cfRNAs and *EV* cfRNAs to discriminate between cancer patients and healthy individuals in a proof‐of‐concept cohort. We sequenced cfRNAs in the plasma samples of LC (*Plasma n* = 19, EV *n* = 19, 18 of them paired) and CRC (*Plasma n* = 23, EV *n* = 19, 19 of them paired) patients (Supporting Information Figure 7). To maximise the sample size, we merged CRC and LC together as a combined cancer group. Based on differential expression analysis between this combined cancer group (*Plasma n* = 42, EV *n* = 38, 37 of them paired) and normal controls (NCs, *Plasma n* = 19, EV *n* = 14, seven of them paired) using the criteria of |log_2_fold‐change| >1 and FDR <.05, we defined a set of cancer‐relevant cfRNAs in both *Plasma* and *EVs* (Supporting Information Figure 17). Interestingly, when we intersected the cancer‐relevant cfRNAs and selective cfRNAs mentioned above, we found that cancer‐relevant cfRNAs accounted for 59.8% (162/271) of the selectively enriched cfRNAs in *Plasma*, whereas they only represented 6.9% (19/274) of the selectively enriched cfRNAs in *EVs*. Therefore, cancer‐relevant cfRNAs appear to be more enriched in *Plasma*’s selective cfRNA fraction (Figure 6A). We also found that enriched functions of these cancer‐relevant *Plasma* cfRNAs were termed as RNA splicing, snRNP signals and so forth (Figure 6B), which were consistent with the enriched pathways of *Plasma* cfRNAs revealed in Figure 5B.

Based on these selectively distributed cancer‐relevant cfRNAs, we endeavoured to discriminate cancer patients from NCs. Although the selective cfRNAs in *Plasma* performed slightly better than those in *EVs* (average area under the receiver operating characteristic curves [AUROC]: .909 vs. .877; Figure 6C and Supporting Information Figure 18), comparable performances were observed between *Plasma* and *EV* cfRNAs when a large number of non‐selective cfRNAs were included as well (average AUROC: .936 vs. .953; Figure 6D and Supporting Information Figure 19). Collectively, these results imply that the purification of EV can reveal distinct cancer signals, but it has a very subtle effect on the accuracy of detection of cancer patients from healthy controls.

We further assessed the potential of cfRNAs (human‐derived only) in *Plasma* and *EV* for classifying CRC from LC. Initially, neither cfRNAs in *Plasma* nor *EV* exhibited strong classification potential (average AUROC: .628 vs. .659; Figure 6E and Supporting Information Figure 20). A recent study revealed that microbe‐derived cfRNAs in human plasma reflect cancer‐type‐specific information.^19^ Based on the RNA abundance of the contamination‐filtered microbe genera, we found the microbial cfRNAs improved the classification of cancer types for both *Plasma* and *EV* cfRNAs (average AUROC: .898 vs. .772; Figure 6E and Supporting Information Figure 20).

Notably, the microbial cfRNAs in *Plasma* performed better than those in *EV*. Consistently, we also found more cancer‐type‐specific features in *Plasma* cfRNAs than in *EV* cfRNAs (Figure 6F). We identified the microbial features recurrently showing differential abundance between CRC and LC in all of the 20 bootstrap samplings. The abundance of top recurrent microbe genera, along with fold‐change and FDRs were illustrated (Figure 6G). For instance, we observed a higher relative abundance of *Methanothrix* in CRC compared to LC using *EV* cfRNA‐seq data. This is consistent with a previous study reporting that *Methanothrix soehngenii* was enriched in gut microbiome of CRC patients.^28^ Meanwhile, many cancer‐relevant viral RNAs in *Plasma* classified cancer types, consistent with the observation of more viral RNAs detected in *Plasma* than in *EVs* (Figure 4G). For instance, *Plasma* cfRNA‐seq data revealed a higher abundance of *alpha‐polyomavirus* and *beta‐polyomavirus*. Supportively, some polyomaviruses were also reported to be detectable in gastrointestinal tract and respiratory aspirates.^29^ These findings suggest that microbe‐derived cfRNAs in *Plasma*, at least in this small cohort with limited sample size, present promising but yet poorly investigated signatures for specific cancer types. Further validation in larger cohorts is required to establish the clinical utility and significance of these preliminary findings.

## 3 DISCUSSION

### 3.1 Technologies utilised and optimised in DETECTOR‐seq

Plasma cell‐free transcriptome remains challenging to study owing to the low quantity and quality of fragmented cfRNAs.^11^ Over‐represented rRNA and mtRNA species,^12^ DNA contamination,^15^ and high cost are still the major issues of cfRNA sequencing. Multiple technologies were included in DETECTOR‐seq to address these issues (Figure 3F). First, DETECTOR‐seq captures fragmented cfRNAs with random priming and template‐switching strategies, which have been proven to be highly efficient in single‐cell RNA‐seq.^30^ Second, the early barcoding protocol of DETECTOR‐seq enables us to prepare cfRNA libraries in a multiplexed manner, thus reducing the volume of required plasma and experimental costs. In fact, DETECTOR‐seq is capable of detecting cfRNAs with a low‐input volume of .2–1 mL plasma with a two to sixfold cost saving compared to existing approaches. Third, with UMIs tagging to cDNAs of RNA fragments, DETECTOR‐seq can accurately quantify the low‐quantity cfRNAs. Fourth, by optimising the procedures of RNA extraction and residual DNA digestion (Supporting Information Figures 3 and 4), DETECTOR‐seq avoids the potential contamination of genomic DNAs. Fifth, DETECTOR‐seq uses CRISPR‐Cas9 technology to deplete rRNA and mtRNA sequences. A CRISPR‐based depletion strategy, DASH^18^ has been utilised in other fields, such as ATAC‐seq,^31^ small RNA‐seq,^32^ bacterial RNA‐seq,^33^ Ribo‐seq^34^ and single‐cell total RNA‐seq.^35^ Here, we applied this CRISPR‐based method to cfRNA sequencing and designed a specific set of sgRNAs for human plasma (Supporting Information Figures 1 and 2). Of note, our sgRNAs target almost the entire length of human rRNAs and mtRNAs, enabling the use of our guides to deplete rRNAs and mtRNAs from any intact or fragmented RNA samples, regardless of the specimen type. This underscores the versatility of our approach beyond plasma samples.

### 3.2 Distinct signatures in *Plasma* versus *EV* cfRNAs

To the best of our knowledge, plasma mainly contains miscellaneous cfRNAs released from alive or apoptotic cells, while RNAs in EV cargos are considered to be actively secreted by cells for functional roles in intercellular communications.^36^ By analysing paired samples of plasma and EVs, this study has provided new insights into distinct cfRNA signatures in plasma versus EVs. We found that plasma cfRNAs are enriched with shorter fragments (20–100 nt) and contain abundant tRNAs, Y RNAs and circRNAs, while EV cfRNAs consist of longer fragments (>100 nt) and are enriched with mRNAs and srpRNAs. In a previous study, Wei et al. analysed extracellular RNA components in the culture medium of glioblastoma cells and found that mRNA is predominantly enriched in vesicles, while non‐vesicular RNP complexes are more enriched with tRNA and Y RNA fragments.^2^ This is consistent with the RNA types enriched in plasma and EVs detected by DETECOR‐seq in this study. It has been proposed that tRNA and Y RNA fragments released by cells in a non‐vesicular form are originally full‐length transcripts, which are cleaved into RNA fragments (tRNA halves and Y RNA fragments) by RNase 1 in the extracellular environment.^37^ Although our results demonstrate that the transcriptomes of plasma and EVs are mainly contributed by blood cells, cfRNA signals from other cell types can also be detected in plasma and EVs. The diversities of cell types of origin in EV cfRNAs are significantly higher than those in plasma cfRNAs, potentially due to the fact that a greater variety of non‐blood cell‐derived RNA signals can be detected in EVs. We found that cfRNAs derived from SINEs, LINEs, LTRs and DNA transposons are more enriched in plasma compared to EVs. Recent study has shown that cancer patients exhibit an enrichment of repeat‐derived cfRNAs, including TEs and other repetitive sequences, in their plasma cell‐free transcriptomes, which can serve as cancer‐specific diagnostic markers.^38^ By identifying the selective RNAs in plasma and EVs, we revealed that plasma is enriched with RNA splicing and associated RNPs, while EVs are enriched in terms associated with DNA binding transcription factor activity, focal adhesion and cell–substrate junction. Plasma and EVs also exhibit distinct immune pathways. These results provide detailed characterisation of plasma and EV transcriptomes, expanding our understanding of cell‐free transcriptomes.

### 3.3 *Plasma* versus *EV* in cancer detection and cancer type classification

In cancer diagnosis, researchers have investigated the potential of plasma cfRNAs in classifying CRC, stomach cancer, liver cancer, LC and oesophageal cancer.^19^ EV cfRNAs have also shown great promise in identifying pancreatic cancer^7^ and prostate cancer^39^ form healthy individuals. However, which cfRNA signals are enriched or lost upon purification of EVs? How do the molecular characteristics of cfRNAs in plasma differ from those in EVs? Which is superior for cancer diagnosis and classification, *Plasma* cfRNAs or *EV* cfRNAs? The answers to these questions are crucial for liquid biopsy researches for clinical and translational application. Our proof‐of‐concept study indicated that purification of EVs provided limited benefit in differentiating cancer patients (colorectal and LC) from healthy individuals, while losing some microbial RNA signals and thereby reducing the ability for cancer type classification. Purification of EVs requires additional experimental procedures and incurs economic costs. Moreover, there is currently no standardised EV purification method suitable for clinical applications. Polyethylene glycol (PEG)‐based precipitation, ultrafiltration, differential ultracentrifugation, size exclusion chromatography, membrane affinity and immunoprecipitation all require a trade‐off between recovery and specificity,^40^ posing significant obstacles to clinical translation and applications. Therefore, as observed in our study, purification of EVs may not be advantageous when clear benefits in cancer diagnosis and classification cannot be achieved. It is important to note that the results of our study need to be validated for their clinical significance in larger scale cohorts.

### 3.4 The relevance of selective microbial signatures

Previous studies have established an approach for cancer diagnosis based on the microbiome analysis of plasma cfDNA.^41^ In this study, we identified some selective microbial signatures in plasma and EV cfRNAs. Furthermore, cancer types can be better classified with microbe‐derived features in plasma cfRNAs than those in EV cfRNAs. Some of these microbial signatures may have relevance. For example, we found that polyomaviruses exhibit higher abundance in plasma of LC patients compared to CRC. Polyomaviruses, a family of DNA viruses, have been investigated for their potential association with human cancers. A member of the alpha‐polyomavirus genus, Merkel cell polyomavirus (MCPyV), has been reported to be associated with Merkel cell carcinoma of the skin.^42^ Studies have also established the association between MCPyV and epidermal growth factor receptor (*EGFR*) mutations in non–small‐cell lung cancer (NSCLC).^43^ The presence of MCPyV DNA has also been significantly correlated with cancer prognosis in subgroups of NSCLC patients with pN0 stage, squamous cell carcinoma or EGFR mutations.^44^ Our exploratory study reveals the potential of discovering microbial signatures associated with cancer occurrence from cfRNA sequencing data generated by DETECTOR‐seq. However, the clinical significance of these microbial signatures still requires further exploration and validation.

### 3.5 Potential utilisation of DETECTOR‐seq in other biofluids

As widely acknowledged, various human body fluids, including plasma, serum, cerebrospinal fluid, saliva, urine, bile, seminal fluid and amniotic fluid, contain cfRNAs.^45^, ^46^, ^47^ The discovery of cfRNAs in these biofluids has also sparked interest in their clinical and translational applications. The cfRNA profiles in different body fluids have been shown to exhibit considerable differences, reflecting the tissues or cells contributing predominantly to the cell‐free transcriptome of the biofluids and their biological functions.^47^ Blood, due to its systemic circulation, has been extensively explored as a valuable source of cfRNAs in diverse conditions. Researchers have already identified the potential of plasma cfRNAs in predicting conditions such as preeclampsia in pregnancy,^4^, ^5^ various cancers^19^ and Alzheimer's disease.^48^ CfRNAs in other body fluids, such as cerebrospinal fluid in brain diseases (Parkinson's disease or brain cancer), urine in prostate or bladder cancer and saliva in oral cancer, may be crucial for liquid biopsy studies in these specific scenarios. In this study, we have demonstrated the capability of DETECTOR‐seq to detect cfRNAs in plasma and EVs. We anticipate that it can serve as a cost‐effective and powerful tool for researchers to explore cfRNAs in other types of body fluids. However, cfRNAs in other biofluids may exhibit certain characteristics that differ from plasma cfRNAs, such as the relatively larger proportion of cfRNAs derived from microbial sources in saliva.^49^, ^50^ Therefore, when further applying DETECTOR‐seq to other biofluids, appropriate modifications may be necessary in library preparation, QC and sequencing data analysis.

### 3.6 Limitations of this study

First, to minimise the influence of cellular RNAs on the low abundance of cfRNAs, we applied a high *g* force (16 000 × *g*) centrifugation to remove cellular debris during plasma preparation. Consequently, a substantial proportion of microvesicles sedimented and were subsequently discarded. And the observed EV cfRNA signatures in this study mainly represented the small EV population ranging from 50 to 200 nm, such as exosomes. Second, while the off‐target effects are minimal, the CRISPR‐Cas9 depletion treatment may still have an impact on the abundance levels of certain pseudogenes, Y RNAs and lncRNAs. Third, while analysing paired plasma samples can increase statistical power, it is important to note that the conclusions regarding the comparison of cfRNAs in *Plasma* and *EVs* for cancer differentiation in this study are still preliminary due to the small sample size. These results serve as a proof‐of‐concept exploration of DETECTOR‐seq's potential for uncovering intriguing insights into real‐world clinical samples. Larger scale cohorts are required to validate these findings and establish their clinical utility. Furthermore, although DETECTOR‐seq offers several advantages compared to other approaches, there is room for further improvement. For instance, the efficiency of random priming in DETECTOR‐seq is influenced by the fragment length of RNAs, which can introduce bias in the library preparation. And DETECTOR‐seq involves several purification steps to eliminate by‐products such as empty library constructs, adapter dimers and excessive primers. These purification procedures tend to retain longer RNA fragments, resulting in the discarding of RNA fragments shorter than 50 nucleotides, along with the by‐products. To obtain a complete spectrum of cfRNAs, including both small and long fragments, DETECTOR‐seq could be modified by incorporating alternative strategies such as poly(A) tailing.^51^, ^52^

## 4 CONCLUSION

In summary, this study introduced a depletion‐assisted cost‐effective cfRNA profiling approach, termed DETECTOR‐seq, which overcomes challenges associated with low quantity and low quality of fragmented cfRNAs, over‐represented rRNAs and mtRNAs, DNA contamination and high costs. Using DETECTOR‐seq, we recapitulated molecular characteristics of *Plasma* and *EV* cfRNAs and identified their distinct human and microbial signatures, thus illustrating the gain and loss of certain cfRNA signals due to EV purification. Our work provides a practical reference for researchers engaged in plasma and EV cfRNA‐based liquid biopsy (Table 1). Moreover, we envision that DETECTOR‐seq would be a useful tool to facilitate further studies in the fields of extracellular RNA biology and plasma or EV cfRNA‐based liquid biopsy, paving the way for advancements in both fundamental research and translational medicine.

**TABLE 1:** Practical reference for cell‐free RNA (cfRNA)‐seq in human plasma.

	*Plasma* cfRNA	*EV* cfRNA
EV purification	No	Yes
Cost of plasma volume, experimental time and reagents^a^	Relatively less	Relatively more
Enriched RNA species	circRNA, tRNA, Y RNA	mRNA, srpRNA
Enriched microbes	Viruses	Intestinimonas and so forth
Diversity of cell‐types‐of‐origin	Relatively low	Relatively high
TE RNAs^b^	Relatively more	Relatively less
Cancer detection	Good (AUC: .94)	Good (AUC: .95)
Cancer‐type‐specific microbes	Relatively more	Relatively less
Cancer type classification	Relatively good (AUC: .90)	Relatively poor (AUC: .77)

Abbreviations: circRNA, circular RNA; EV, extracellular vesicle; mRNA, messenger RNA; srpRNA, signal recognition particle RNA; TE, transposable element.

^a^ Different cost is due to the EV purification step.

^b^ Cell‐free RNAs derived from transposable elements.

## 5 MATERIALS AND METHODS

### 5.1 Cohort design

Seventy‐five participants, including patients with CRC (*n* = 24), LC (*n* = 20) and healthy controls (*n* = 31), were enrolled in this study. Samples were obtained from November 2018 to January 2022. Individuals with CRC, LC and healthy controls were recruited from Peking University First Hospital. All samples were used for matched detection of plasma and EV cfRNAs. After QC of RNA samples and sequencing data (Supporting Information Figure 7), we obtained a total of 113 datasets, including 19 plasma and 14 EV datasets (seven of which were paired) from 26 healthy donors, 23 plasma and 19 EV datasets (19 of which were paired) from 23 CRC samples and 19 plasma and 19 EV datasets (18 of which were paired) from 20 LC samples. The characteristics of participants in this study were summarised (Supporting Information Table 9).

### 5.2 Sample collection

Peripheral whole blood samples were collected in EDTA‐coated vacutainer tubes for each participant. Blood samples of patients with cancer were collected before any treatment of surgery, chemotherapy or neoadjuvant chemotherapy. Within 2 h after blood collection, blood samples were centrifuged at 1900 × *g* for 30 min at room temperature. Plasma was separated and then centrifuged at 16 000 × *g* for another 10 min at 4°C to remove cellular debris. All plasma samples were aliquoted and stored at −80°C until analysis.

### 5.3 RNA extraction of plasma and extracellular vesicles

cfRNAs were extracted from 1 mL of plasma using QIAzol Lysis Reagent (Qiagen, 79306) according to the manufacturer's instructions. The upper, aqueous phase containing cfRNAs was mixed with 1 volume of ethanol (95%–100%) and then added to the Zymo‐Spin column (Zymo, R1016) for RNA binding. Samples were subsequently washed, eluted and treated with DNase I (TaKaRa, 2270A) for 20 min at 37°C. Following residual DNA digestion, cfRNAs were then purified and concentrated into 6 µL using an RNA Clean and Concentrator‐5 kit (Zymo, R1016). Plasma EVs were purified by a membrane‐affinity approach using an exoRNeasy Midi Kit (Qiagen, 77144) following the manufacturer's instructions.^53^ EVs were eluted with 400 µL of elution buffer and characterised by TEM and nanoparticle tracking analysis (NTA). For RNA isolation, EVs were lysed on the exoRNeasy column using QIAzol Lysis Reagent, and EV RNAs were extracted and purified using Zymo‐Spin column as mentioned above.

### 5.4 Optimisation of cell‐free RNA extraction and residual DNA digestion

RNA extraction is one of the most critical steps for low‐input RNA‐seq. To this end, we compared three cfRNA extraction approaches, including QPCB (QIAzol lysis, phenol–chloroform extraction and column binding), Norgen (Plasma/Serum Circulating and Exosomal RNA Purification Kit) and QPIP (QIAzol lysis, phenol–chloroform extraction and isopropanol precipitation). QPCB was considered the best approach for cfRNA extraction (Supporting Information Figure 3).

In previous reports, DNA contamination has been emphasised as a hinder to the cfRNA study.^15^ Therefore, we examined two major residual DNA digestion approaches: On‐column versus In‐buffer (On‐column: residual cell‐free DNA was digested on the spin column during RNA extraction; In‐buffer: DNA was digested in the aqueous buffer after RNA extraction). We observed a significantly higher human genome mapping ratio and exonic read ratio with In‐buffer DNA digestion than On‐column approach (*p* value <.0001, Wilcoxon rank sum test; Supporting Information Figure 4), suggesting In‐buffer DNA digestion was more effective to a certain extent. DETECTOR‐seq was carried out in the following assays with RNAs extracted using QPCB and residual DNA digested with an In‐buffer approach unless specified.

### 5.5 ERCC RNA Spike‐In Mix

To evaluate the quantitative accuracy of DETECTOR‐seq in measuring RNA abundance, we added 1 µL of a 1:10 000 dilution of ERCC Spike‐In RNA Mix 1 (Catalogue Number: 4456740) to the total cfRNAs isolated from 1 mL of plasma samples. The concentration information for these Spike‐In RNAs can be found in Thermo Fisher's website (https://assets.thermofisher.cn/TFS‐Assets/LSG/manuals/cms_095046.txt). We adjusted the original concentration of ERCC Spike‐In RNA in Mix 1 (attomoles/µL) by dividing it by 10 000 (dilution ratio) and 1E18 (attomoles to moles), and then multiplied it by 6.022E23 (molecules per mole; Avogadro's number) to determine the number of molecules added to our cfRNA samples. The Pearson correlation coefficient between the expected abundance of ERCC Spike‐In RNAs and the detected levels by DETECTOR‐seq was utilised to assess the quantitative accuracy. A total of four plasma cfRNA samples from LC patients were employed as replicates, and we observed a consistently high correlation between the expected and observed transcript abundance levels. One representative result was presented in Supporting Information Figure 5F. After confirming the quantitative accuracy of DETECTOR‐seq, we did not add ERCC Spike‐In Mix in the other samples in our cohort.

### 5.6 Reverse transcription

CfRNAs were captured using random primers with a unique sample barcode and then reverse transcribed with SMARTScribe reverse transcriptase (Clontech, 639538) and template‐switching oligos tagging 8‐nt UMI sequences. Sample barcodes were designed in R using the DNABarcodes package.^54^ We generated barcodes with a length of four nucleotides and a minimum Hamming distance of 3 and filtered self‐complementary sequences, triplets and sequences that have an unbalanced ratio of bases G or C versus A or T. PEG 8000 (Beyotime, R0056‐2 mL) was used as molecular crowding reagent to further improve the efficiency of reverse transcription reaction.^55^ The 20‐µL reaction mixture was incubated at 42°C for 90 min with a heat inactivation step at 70°C for 10 min. Primers for the reverse transcription of DETECTOR‐seq were shown in Supporting Information Table 3.

### 5.7 Quantitative PCR analysis

The total abundance level of *Plasma* or *EV* cfRNAs was assessed by amplifying a fragment from the human gene of *ACTB* spanning the exon–exon junction (ACTB‐ee). The level of residual DNA contamination was measured by amplifying a short fragment from *ACTB* within intron regions (ACTB‐i). We measured the microbiome contamination by the threshold cycle (Ct) value difference of the ACTB‐ee and the bacterial 16S ribosomal RNA V4 fragment (16S‐V4). The 2.5 µL of cDNA template was amplified in a final volume of 20 µL using the FastFire qPCR PreMix (SYBR Green; TIANGEN, FP207). Samples with low RNA content (Ct of ACTB‐ee >32) or high DNA contamination (Ct of ACTB‐i <35), or high bacterial contamination (ΔCt (ACTB‐ee–16S‐V4) >5) were excluded for further analysis. We summarised the QC primers for *Plasma* and *EV* cfRNA samples (Supporting Information Table 1).

### 5.8 Design of guide RNAs

To remove highly abundant sequences (rRNAs and mtRNAs) in the cfRNA library of human plasma, we designed 302 and 315 high‐quality sgRNAs specifically targeting the ribosomal and mitochondrial RNA sequences (Supporting Information Figure 1). The sgRNAs were selected and filtered by DASHit^56^ and Benchling (https://www.benchling.com/crispr) based on the following criteria: (1) off‐target score (specificity score) and on‐target score (sensitivity score); (2) poorly structured sgRNAs were excluded; (3) cover approximately every 50 bp over the target sequences. First, candidate guides in rRNA (4324) and mtRNA (1907) were outputted by scanning all the protospacer adjacent motif (PAM) sequences. Meanwhile, the transcriptome of hg38 (excluded rRNA and mitochondrial RNA) was also scanned for all CRISPR sites as an off‐target list. Second, we excluded off‐target guides and filtered poorly structured guides, including (a) G/C frequency too high (>15/20) or too low (<5/20); (b) Homopolymer: more than five consecutive repeated nucleotides; (c) Dinucleotide repeats: the same two nucleotides alternate for >3 repeats; (d) Hairpin: complementary subsequences near the start and end of a binding site, causing a hairpin. And we got 1191 rRNA guides and 1425 mtRNA guides as qualified guides. Next, we downloaded guides designed by Benchling to score our guides and remove redundant guides (overlapped with each other), and kept guides with higher on‐target and off‐target scores. Thus, we got a pool of filtered guides. Finally, we manually added some guides to cover the non‐guide regions and Single Nucleotide Polymorphism (SNP) sites and obtained a final sgRNA pool containing 302 guides targeting rRNA sequences and 315 guides targeting mtRNA sequences. The DNA templates of final sgRNAs were synthesised through a one‐step PCR using two paired primers to achieve the addition of T7 promoter and guide RNA scaffold sequences (primers for preparation of sgRNA DNA templates were shown in Supporting Information Table 2). The final sgRNAs were in vitro transcribed using T7 RNA polymerase (NEB, E2050) and stored at −80°C.

### 5.9 DETECTOR‐seq library preparation

The 17.5 µL of remaining samples with similar cDNA content (ΔCt of ACTB‐ee <1) were pooled for 3‐, 4‐ or 5‐plex library preparation. The pooled cDNAs were pre‐amplified using SeqAmp DNA Polymerase (Clontech, 638509) with the following PCR setup: initial denaturation at 94°C for 1 min, denaturation at 98°C for 15 s, annealing at 55°C for 30 s, elongation at 68°C for 30 s and final elongation at 68°C for 10 min. Pre‐amplification was repeated for six cycles, and the DNA was cleaned and size selected using Hieff NGS DNA selection Beads (Yeasen, 12601ES56) at a ratio of 1:.8 of DNA to beads twice. The DNA was eluted with the 20 µL CRISPR‐Cas9 reaction mix consisting of 300 ng sgRNAs for rRNA sequences, 40 ng sgRNAs for mtRNA sequences, 1× NEBuffer3.1 and 1 µM Cas9 Nuclease, *S. pyogenes* (NEB, M0386). The 20‐µL reaction mixture was incubated at 37°C for 60 min and heat inactivated at 65°C for 5 min. Following the depletion of DNA fragments derived from rRNAs and mtRNAs, the remaining DNA samples with complete library structure were amplified for 16–18 cycles depending on the initial input. The final clean‐up was conducted at a ratio of 1:1 of DNA to beads. The library concentration was measured using a Qubit dsDNA HS Assay Kit (Thermo Fisher, Q32854), and the size distribution of the library was assessed using an Agilent 2100 Bioanalyzer with a High‐Sensitivity DNA analysis kit (Agilent, 5067‐4626). DETECTOR‐seq libraries were sequenced on an Illumina HiSeqX platform to a depth of 10 million 150 bp paired‐end reads per sample. Primers for PCR amplification of DETECTOR‐seq were shown in Supporting Information Table 4.

### 5.10 Sequencing data processing

Raw sequencing data were demultiplexed using sabre (https://github.com/najoshi/sabre) according to sample barcodes. UMI sequences were extracted using UMI‐Tools.^57^ Adapters were removed by cutadapt and read pairs with an average quality score below 30 in either read were removed. The remaining read pairs were then sequentially mapped to ERCC's spike‐in sequences, NCBI's UniVec sequences, human rRNA sequences, human mtRNA sequences, human genome (hg38) and circular RNA using STAR (version 2.5.3a_modified).^58^ This means that reads aligned to one set of sequences were not used for alignment to the subsequent set of sequences. The UMI‐Tools package was used to remove duplicated reads caused by PCR amplification. An aligned read pair was assigned to an RNA type if at least one of the mates overlapped with the corresponding genomic regions. In this way, the aligned reads were sequentially assigned to mRNA, lncRNA, pseudogene, tRNA, srpRNA, snoRNA, snRNA, Y_RNA, misc_RNA and other exon regions with HTSeq package^59^ according to the GENCODEv38 annotation. Unmapped reads were classified using kraken2^60^ to obtain microbe (including bacterial, archaeal and viral) genus abundance. Potential contaminations in genera were filtered before downstream analysis as in previously published work.^19^ We summarised all the datasets for the development, validation and application of DETECTOR‐seq in Supporting Information Table 8.

### 5.11 Quality control (sample filtering and gene filtering)

To filter low‐quality DETECTOR‐seq datasets, the following QC criteria were used (Supporting Information Figure 7): (1) raw reads >4 M; (2) clean reads (reads remained after trimming low‐quality and adapter sequences) >3.8 M; (3) ribosomal RNA reads <20%; (4) mitochondrial RNA reads <20%; (5) genome‐aligned reads >2 M; (6) de‐duplicated RNA reads >.1 M. Next, we retained genes with TPM >1 in at least 50% of samples or filtered genes by filterbyExpr of edgeR package.^61^

### 5.12 Differential expression and functional enrichment analysis

The count matrix of gene expression or microbe genus abundance was normalised using the trimmed mean of M‐values (TMM) method in the edgeR package.^61^ Differential expression analysis was conducted using a quasi‐likelihood method with FDR <.1 to identify RNAs showing a selective distribution in paired *Plasma* and *EV* samples and to identify differentially expressed genes (DEGs; |log_2_fold‐change| >1 and FDR <.05) between cancer patients and NCs. We conducted the Gene Set Enrichment Analysis (GSEA) and functional enrichment analysis of Gene Ontology (GO) using the R package clusterProfiler.^62^ The genes are ordered based on log2FoldChange and *p* value, and the enriched GO terms were determined using the gseGO function. GO terms enriched in *Plasma* were filtered with normalised enrichment score (NES) cut‐off of <−1 and *p*.adjust cut‐off of <.1. Conversely, GO terms enriched in *EVs* were filtered with NES cut‐off of >1 and *p*.adjust cut‐off of <.1.

### 5.13 Enrichment analysis of RBP binding motifs/sites

After identifying selective RNAs showing significantly differential abundance between *Plasma* and *EV* transcriptomes, we conducted an enrichment analysis of RBP binding motifs/sites using MEME SEA.^63^ We first created a gene‐wise ‘RBP binding hotspot’ sequence set by expanding annotated exon junction sites upstream and downstream by 20 nt and combined with 5′ UTR and 3′ UTR regions (GENCODE v38), as these regions were reported to be frequently bound by RBPs.^64^ Background sequences were extracted from 500 random subsets of cfRNAs whose abundance showed no significant difference between *Plasma* and *EV* (FDR >.1). Database files of RBP binding motifs/sites for enrichment analysis were annotated from our previous research.^64^ Finally, top enriched RBPs (ranked by *E* value) were annotated and summarised, and sequence logo images were created from POSTAR3 database^64^ using WebLogo.^65^

### 5.14 Deconvolution of cell types of origin

We applied Nu‐SVR to deconvolve the fractions of cell‐type‐specific RNAs based on Tabula Sapiens version 1.0 (TSP), a multiple‐donor whole‐body cell atlas spanning 24 tissues and organs as previously reported.^23^

### 5.15 Cancer classification

We normalised and scaled gene expression and genus abundance for evaluating the cancer‐differentiating capacity of human and microbial features in *Plasma* and *EV* cfRNAs. All of the 61 *Plasma* and 52 *EV* DETECTOR‐seq datasets passed QC were used, thus including as many cases as possible in the training and test sets. Most of the cancer samples were paired between *Plasma* and *EV* (CRC samples: *Plasma* 23, *EV* 19, 19 of them were paired; LC samples: *Plasma* 19, *EV* 19, 18 of them were paired; NC samples: *Plasma* 19, *EV* 14, seven of them were paired). The data were trained and tested with bootstrapping sampling, which was randomly repeated 20 times. For human RNAs, a quasi‐likelihood method was used for the differential expression analysis in each bootstrapping procedure. In Figure 6C, differentially expressed features with |log_2_fold‐change| >1 and FDR <.05 overlapped with RNAs that were enriched in *Plasma* or *EV* (defined in Figure 5A) were further used to fit a random forest classifier. In Figure 6D, we selected the top 200 features ranked by FDR in each bootstrapping procedure. For microbial RNAs, we selected all of the microbe genera with |log_2_fold‐change| >1 and FDR <.1 in each bootstrapping procedure. For the combination of human RNAs and microbial RNAs, we combined human gene expression and genus abundance and selected the top 200 features ranked by FDR. The AUROC was calculated from the final probability using the pROC^66^ package in R.

### 5.16 Cost estimation

The cost for cfRNA library preparation of DETECTOR‐seq was determined using the sum of the price for each component used in our protocol. The price of SMARTer Stranded Total RNA‐Seq Kit v2‐Pico Input Mammalian (TaKaRa, 634413) was searched on the official website of TaKaRa for the estimation of SMARTer‐seq. The cost of phospho‐RNA‐seq was estimated using T4 polynucleotide kinase (NEB, M0201S) and TruSeq small RNA kit (Illumina, RS‐200‐0012). In the case of SILVER‐seq, there was no publicly available step‐by‐step protocol, thus the cost of SILVER‐seq was estimated by the Ovation SoLo RNA‐Seq Kit (NuGEN, 0500‐96). In all cases, the prices listed in Supporting Information Tables 6 and 7 included sales tax. Because the costs of SMARTer‐seq, phospho‐RNA‐seq and SILVER‐seq were estimated using commercial kits (including additional selling costs and profits), for a fair comparison, we determined the cost of DETECTOR‐seq as twice the calculated price.

## AUTHOR CONTRIBUTIONS

Hongke Wang, Qing Zhan and Zhi John Lu conceived and designed the project; Hongke Wang and Qing Zhan developed DETECTOR‐seq; Hongke Wang, Qing Zhan, Meng Ning and Shaozhen Xing generated the datasets; Hongjie Guo, Shuai Zuo, Shanwen Chen and Pengyuan Wang collected the clinical samples; Qing Zhan and Hongke Wang conducted the analyses; Zhi John Lu, Pengyuan Wang and Mengtao Li were responsible for supervision; all authors wrote the manuscript; Hongke Wang, Qing Zhan, Xuefeng Xia and Zhi John Lu revised the manuscript; all authors read and approved the final manuscript.

## CONFLICT OF INTEREST STATEMENT

A patent application on the described technology has been filed by Hongke Wang and Zhi John Lu. Other authors declare no conflicts of interest.

## ETHICS STATEMENT

This study was approved by the institutional review board of Peking University First Hospital (2018‐15). Informed consent was obtained from all patients.

## Supporting information

Supporting Information

## Data Availability

Data generated with DETECTOR‐seq are available at the Gene Expression Omnibus under accession number GSE216561. For benchmarking, we used the following datasets: GSE126049 (phospho‐RNA‐seq), GSE131512 (SILVER‐seq) and GSE174302 (SMARTer‐seq).
